# Marginal zone lymphoma international prognostic index: a unifying prognostic index for marginal zone lymphomas requiring systemic treatment

**DOI:** 10.1016/j.eclinm.2024.102592

**Published:** 2024-04-11

**Authors:** Luca Arcaini, Côme Bommier, Juan Pablo Alderuccio, Michele Merli, Nicole Fabbri, Maria Elena Nizzoli, Matthew J. Maurer, Vittoria Tarantino, Simone Ferrero, Sara Rattotti, Annalisa Talami, Roberta Murru, Arushi Khurana, Raphael Mwangi, Marina Deodato, Emanuele Cencini, Francesca Re, Carlo Visco, Andrew L. Feldman, Brian K. Link, Marcia Torresan Delamain, Michele Spina, Ombretta Annibali, Alessandro Pulsoni, Andrés J.M. Ferreri, Caterina Cecilia Stelitano, Elsa Pennese, Thomas M. Habermann, Luigi Marcheselli, Sunwoo Han, Isildinha M. Reis, Marco Paulli, Izidore S. Lossos, James R. Cerhan, Stefano Luminari

**Affiliations:** a Department of Molecular Medicine, University of Pavia, Pavia, Italy; b Division of Hematology, Fondazione IRCCS Policlinico San Matteo, Pavia, Italy; c Hemato-Oncology Department, DMU DHI, Hôpital Saint Louis, Paris, France; d Department of Quantitative Health Sciences, Mayo Clinic, Rochester, MN, USA; e Division of Hematology, Department of Medicine, Sylvester Comprehensive Cancer Center, University of Miami Miller School of Medicine, Miami, FL, USA; f Division of Hematology, University Hospital Ospedale di Circolo e Fondazione Macchi-ASST Sette Laghi, University of Insubria, Varese, Italy; g Division of Hematology, Azienda Unità Sanitaria Locale – IRCCS, Reggio Emilia, Italy; h Clinical and Experimental Medicine Doctorate School, Università degli Studi di Modena e Reggio Emilia, Italy; i Division of Hematology, Mayo Clinic, Rochester, MN, USA; j Division of Hematology, Azienda Ospedaliera Ospedali Riuniti Villa Sofia-Cervello, Palermo, Italy; k Division of Hematology, Department of Molecular Biotechnologies and Health Sciences, University of Torino, and AOU “Città della Salute e della Scienza di Torino”, Torino, Italy; l Hematology and Stem Cell Transplantation Unit, Ospedale Oncologico A. Businco, ARNAS G. Brotzu, Cagliari, Italy; m Division of Hematology, Niguarda Cancer Center, ASST Grande Ospedale Metropolitano Niguarda, Milano, Italy; n Division of Hematology, Azienda Ospedaliera Universitaria Senese and University of Siena, Siena, Italy; o Division of Hematology, Azienda Ospedaliero-Universitaria di Parma, Parma, Italy; p Division of Hematology, San Bortolo Hospital, AULSS 8 Berica, Vicenza, Italy; q Department of Laboratory Medicine and Pathology, Mayo Clinic, Rochester, MN, USA; r Department of Internal Medicine, University of Iowa Hospitals, Iowa City, IA, USA; s Faculty of Medical of Minas Gerais, Feluma, Brazil for Faculty of Medical of Minas Gerais, Belo Horizonte, Brazil; t Division of Medical Oncology and Immune-Related Tumors, Centro di Riferimento Oncologico di Aviano (CRO) IRCCS, Aviano, Italy; u Division of Hematology, Stem Cell Transplantation, University Campus Bio-Medico, Roma, Italy; v Division of Hematology, Sapienza University – Polo Pontino, Department of Translational and Precision Medicine, S.M. Goretti Hospital, Latina, Italy; w Lymphoma Unit, IRCCS San Raffaele Scientific Institute, and University Vita-Salute San Raffaele, Milano, Italy; x Division of Hematology, Grande Ospedale Metropolitano, Bianchi Melacrino Morelli, Reggio Calabria, Reggio Calabria, Italy; y Division of Hematology, Ospedale Spirito Santo, Pescara, Italy; z Fondazione Italiana Linfomi ETS, Italy; aa Biostatistics and Bioinformatics Shared Resource, Sylvester Comprehensive Cancer Center, University of Miami Miller School of Medicine, Miami, FL, USA; ab Department of Public Health Sciences, University of Miami Miller School of Medicine, Miami, FL, USA; ac Division of Pathology, Fondazione IRCCS Policlinico San Matteo, Pavia, Italy; ad Department CHIMOMO, University of Modena and Reggio Emilia, Modena, Italy

**Keywords:** Marginal zone lymphoma, Prognosis

## Abstract

**Background:**

Marginal zone lymphomas (MZL), comprised of three unique but related subtypes, lack a unifying prognostic score applicable to all the patients in need for systemic chemotherapy and/or immunotherapy.

**Methods:**

Patients from the prospective NF10 study (NCT02904577) with newly diagnosed MZL and receiving frontline systemic therapy at diagnosis or after observation were used to train a prognostic model. The primary endpoint was progression-free survival (PFS) from start of treatment. The model was externally validated in a pooled analysis of two independent cohorts from the University of Iowa and Mayo Clinic Molecular Epidemiology Resource and the University of Miami.

**Findings:**

We identified 501 eligible patients. After multivariable modeling, lactate dehydrogenase (LDH) above upper normal limit, hemoglobin <12 g/dL, absolute lymphocyte count <1 × 10^9^/L, platelets <100 × 10^9^/L, and MZL subtype (nodal or disseminated) were independently associated with inferior PFS. The proposed MZL International Prognostic index (MZL-IPI) combined these 5 factors, and we defined low (LRG, 0 factors, 27%), intermediate (IRG, 1–2 factors, 57%) and high (HRG, 3+ factors, 16%) risk groups with 5-y PFS of 85%, 66%, and 37%, respectively (c-Harrell = 0.64). Compared to the LRG, the IRG (Hazard Ratio [HR] = 2.30, 95% CI 1.39–3.80) and HRG (HR = 5.41, 95% CI 3.12–9.38) had inferior PFS. Applying the MZL-IPI to the pooled US cohort (N = 353), 94 (27%), 192 (54%), and 67 (19%) patients were classified as LRG, IRG, and HRG, respectively, and the model was validated for PFS (log-rank test p = 0.0018; c-Harrell = 0.578, 95% CI 0.54–0.62). The MZL-IPI was also prognostic for OS in both the training and the external validation sets.

**Interpretation:**

MZL-IPI is a new prognostic score for use in all patients with MZL considered for systemic treatment.

**Funding:**

The MER was supported by P50 CA97274 and U01 CA195568.

**Research in context**

**Evidence before this study**

Studies to date have mainly focused on defining prognostic factors for specific MZL subtypes, with the development of specific-entity scores such for SMZL and ENMZL (Mucosa-Associated Lymphoid Tissue Lymphoma International Prognostic Index, MALT-IPI and revised MALT-IPI). Application of existing indices (IPI, FLIPI, MALT-IPI) to a cohort of non-follicular indolent lymphomas found that MALT-IPI was the most useful in terms of distribution of risk categories and prognostic discrimination. However, no index has been specifically developed for MZLs as a single group. Such an index would have utility, as most recent clinical trials enroll the entire spectrum of MZL. Furthermore, a unifying prognostic score would also allow to account for a group of MZL patients who show widespread disease without clear primary splenic, nodal or extranodal origin. It should be noted that the existence of disseminated cases and the prognostic impact of widespread disease in ENMZL have been previously reported but these cases have never been included in previous subtype specific studies or are hardly identifiable within published series of MZL.

**Added value of this study**

MZL-IPI represents a step forward in the prognostic assessment of marginal zone lymphoma.

The MZL-IPI is prospectively constructed using marginal zone lymphoma patients with all the clinical subtypes and integrates histological, clinical and laboratory parameters specifically selected and validated in the entire spectrum of marginal zone lymphomas.

The score has been validated in an independent US cohort of patients with marginal zone lymphoma.

Importantly, age was not included in the MZL-IPI, which represents a significant conceptual difference in comparison to other models and may help to better manage an indolent disease such as marginal zone lymphoma in the chemo-free era without age limitations to systemic treatment.

**Implications of all the available evidence**

The MZL-IPI is not intended to be used as a predictive score to support clinical and therapeutic decisions for the individual patient, but it represents an important tool to aid clinical development and to allow improvement in clinical trial designs, results interpretation, and cross trial comparisons.

## Introduction

Marginal zone lymphomas (MZL) are the third most common type of B-cell non-Hodgkin lymphoma (NHL), accounting for about 7% of all NHLs,^1^ and include extranodal marginal zone lymphoma (ENMZL), nodal marginal zone lymphoma (NMZL), and splenic marginal zone lymphoma (SMZL).^2^^,^^3^ MZLs are characterized by slow growth, often do not require immediate therapy, and when treatment is needed, excellent response and progression-free survival (PFS) rates are achieved. For many years, management of MZLs has been derived from strategies used in follicular lymphoma or chronic lymphocytic leukemia. However, MZL exhibits meaningful differences in biology and clinical characteristics with respect to other lymphomas, showing that it represents a unique disease that requires a dedicated therapeutic approach.^2^ While overall outcomes are favorable, with 10-year overall survival (OS) of about 80%,^4^, ^5^, ^6^, ^7^, ^8^, ^9^, ^10^ there is significant heterogeneity among MZL patients overall and within each MZL subtype.^4^^,^^11^

Studies to date have mainly focused on defining prognostic factors for specific MZL subtypes, with the development of specific-entity scores such for SMZL^12^^,^^13^ and ENMZL (Mucosa-Associated Lymphoid Tissue Lymphoma International Prognostic Index, MALT-IPI and revised MALT-IPI).^14^^,^^15^ Application of existing indices (IPI, FLIPI, MALT-IPI) to a cohort of non-follicular indolent lymphomas found that MALT-IPI was the most useful in terms of distribution of risk categories and prognostic discrimination.^16^ However, no index has been specifically developed for MZLs as a single group. Such an index would have utility, as most recent clinical trials enroll the entire spectrum of MZL.^17^^,^^18^ Furthermore, a unifying prognostic score would also allow to account for a group of MZL patients who show widespread disease without clear primary splenic, nodal or extranodal origin. It should be noted that the existence of disseminated cases^19^ and the prognostic impact of widespread disease in ENMZL have been previously reported^20^ but these cases have never been included in previous subtype specific studies or are hardly identifiable within published series of MZL.

We used data from patients prospectively enrolled in the Fondazione Italiana Linfomi (FIL) NF10 observational study to develop a prognostic model for the entire spectrum of MZL. We then conducted an independent validation, pooling two cohorts of MZL patients from the University of Iowa and Mayo Clinic Molecular Epidemiology Resource (MER) and the University of Miami.

## Methods

The prognostic model was developed in the NF10 (NCT02904577), a prospective observational study of newly diagnosed indolent nonfollicular lymphomas (INFL) patients consecutively enrolled from July 2010 through May 2019 by 65 centers in Europe and South America (list of centers and inclusion criteria of NF10 study are reported in the Data Supplement, Sections A and B). An operational approach to MZL grouping and to define disseminated MZL (dissMZL) is provided in the Data Supplement, Section B and Supplementary Table S1): briefly, disseminated MZL (dissMZL) included cases for which it was not possible to identify a clear site of origin and was defined by the co-presence of more than one site among spleen, and/or lymph nodes, and/or extranodal disease, and/or peripheral blood and/or bone marrow involvement.

This study was conducted on the population of the NF10 study with the main aim of developing a prognostic score for patients with MZL. The final analysis cohort was restricted to MZL patients who started a systemic therapy, with the inclusion of patients who were treated at registration and those who received systemic treatment after an initial observation period. Allowed treatment included the use of chemotherapy, immunotherapy, or both. Patients who were initially treated with anti-infectious agents or with local therapies could be enrolled only if subsequently treated with systemic anti-lymphoma therapies. For all eligible patients, clinical and laboratory data were collected at the time of starting systemic treatment, which was considered as the index date for all subsequent study evaluations. Histologic diagnosis was required on tissue or on bone marrow biopsy and was based on local assessment.

### Ethics statement

The study was conducted in accordance with the Declaration of Helsinki Ethical Principles and Good Clinical Practices and was approved at each site by an ethics committee. Signed consent form was mandatory for all enrolled patients. We conducted the study in compliance with STARD-2015 guidelines (equator-network.org).

### Statistical analysis

The primary endpoint of this prognostic study was PFS, which was calculated from the time of systemic treatment start to the date of subsequent progression, or death due to any cause. The secondary endpoint was OS, which was defined from date of systemic treatment to date of death for any cause. Patients without events for both PFS and OS were censored at time of last follow-up.^21^ PFS and OS were estimated by the method of Kaplan–Meier. The log-rank test was used to compare different groups and hazard ratios (HR) and 95% confidence interval (95%CI) were estimated from Cox proportional hazard (PH) regression^22^ either in univariable or multivariable models.

To build an accurate and useful prognostic model we selected commonly available clinical variables, applying previously reported cutpoints and categories to continuous variables. Model development occurred on the complete case set with non-missing data for 12 covariates. The final multivariable Cox model was selected by step-by-step likelihood ratio test, starting from a cohort with full complete cases and considering covariates with p-value < 0.50 in univariable analysis and including in the final model the covariates based on statistical (likelihood ratio test and Akaike information criterion, AIC)^23^ and clinical considerations. The proportionality of risk of the multivariable Cox PH model was checked by means of Grambsch–Therneau test on scaled Schoenfeld residuals^24^ and the model performance was analyzed using the likelihood displacement method.^25^ All reported p-values were two-sided.

A prognostic score was then obtained by assigning a weight to each variable retained in the final Cox model considering the z-score (Wald test). The final score was then defined as the sum of the weights. Based on the evaluation of the Kaplan–Meier curves and Cox model and considering HR of individual or aggregated risk groups, the final prognostic score has been defined. The final model has been internally validated using bootstrap techniques to evaluate the discriminant power (C-index), shrinkage factor (check for overfitting), and calibration (comparison between predicted and actual survival).^26^

A sensitivity analysis was performed to check the stability of the Cox PH model and MZL-PI score with two approach: 1) after multiple imputation (MI) by means of chained equations and applying the Rubin’s rules to combine estimate coefficients across the M imputed datasets; 2) considering the censored patients with follow-up <24 months have been treated a) as competing events and fitting the model by Fine–Gray approach, b) as alive patients with follow-up of 96 months and c) as failures at time of censoring (details in Data Supplement, Section F).

To validate the model, we applied the same inclusion criteria, subtype classification algorithm (including defining dissMZL), and model parameters from the NF10 analysis to an independent pooled cohort of MZL patients from the University of Iowa/Mayo Clinic Lymphoma Specialized Program of Research Excellence (SPORE) Molecular Epidemiology Resource (MER)^27^ and an institutional cohort from the University of Miami, FL, USA (Data Supplement, Section C).^28^

### Role of the funding source

The study was funded by the Fondazione Italiana Linfomi. The funder of the study had no role in study design, data collection, data analysis, data interpretation, or writing of the report. The corresponding authors (Luca Arcaini and Stefano Luminari) had full access to all of the study data and took final responsibility for the decision to submit for publication”.

## Results

Overall, 790 MZL patients were identified from the 1717 patients prospectively enrolled in the NF10 study by 65 centers in Italy and South America. Among them systemic treatment was started in 501 patients including 435 patients who were treated immediately after diagnosis and 66 who started treatment after an initial period of watch and wait: for this latter group median time to treatment start was 18 months (range 4–58; 55% ≤ 24 months) (Data Supplement, Supplementary Fig. S1 and Table 1); 92% of patients were treated with regimens containing anti-CD20 monoclonal antibody (10% rituximab monotherapy, 82% rituximab combined with chemotherapy). After a median follow-up of 61 months (range 1–114 months) from diagnosis, in treated patients we observed 150 events for PFS including 122 progressions and 28 deaths. The 5-y PFS was 67% (95%CI, 62–71%) (Data Supplement, Supplementary Fig. S2a). No difference in terms of PFS was observed comparing the group of patients who received immediate or delayed treatment (Data Supplement, Supplementary Fig. S2b). The 5-y OS for the treated patients was 86% (95%CI 82–89%) (Data Supplement, Supplementary Fig. S3); overall, 68 patients died, including 30 deaths due to lymphoma progression, 9 due to second cancer and 6 due to infection.

**Table 1:** Characteristics of 501 patients with marginal zone lymphoma at time of treatment.

Group	Treated n (%)	Treated after W&W	p	Overall
	n = 435	n = 66		n (%)
Age				
<70 years	259 (60)	28 (42)	0.011	287 (57)
≥70 years	176 (40)	38 (58)		214 (43)
Stage				
I–II	90 (21)	5 (8)	0.007	95 (19)
III–IV	338 (79)	61 (92)		399 (81)
Missing	7	–		7
Bone marrow				
Negative	183 (44)	5 (13)	<0.001	188 (42)
Positive	229 (56)	35 (87)	<0.001	264 (58)
Missing	23	26		49
ENS				
0–1	358 (80)	53 (91)	0.046	403 (82)
>1	85 (20)	5 (9)		90 (18)
Missing	–	8		8
Nodal size				
≤6 cm	340 (88)	48 (87)	0.826	388 (88)
>6 cm	46 (12)	7 (13)		53 (12)
Missing	49	11		60
ECOG-PS				
0–1	401 (93)	44 (88)	0.249	445 (93)
>1	30 (7)	6 (12)		36 (7)
Missing	4	16		20
LDH				
≤ULN	293 (69)	36 (63)	0.449	329 (68)
>UNL	133 (31)	21 (37)		154 (32)
Missing	9	9		18
B2M				
≤ULN	132 (37)	9 (21)	0.042	171 (36)
>UNL	221 (63)	33 (79)		254 (64)
Missing	82	24		106
Hemoglobin				
≥12 mg/dL	256 (59)	29 (53)	0.387	285 (58)
<12 g/dL	178 (41)	26 (47)		204 (42)
Missing	1	11		12
ALC				
≥1 10^9^/L	345 (80)	36 (71)	0.103	381 (79)
<1 10^9^/L	84 (20)	15 (29)	0.103	99 (21)
Missing	6	15		21
Platelets				
≥100 10^9^/L	373 (86)	45 (82)	0.417	418 (85)
<100 10^9^/L	61 (14)	10 (18)		71 (15)
Missing	1	11		12
MZL subtype			<0.001	
ENMZL	188 (43)	9 (14)		197 (39)
SMZL	128 (29)	38 (57)		166 (33)
NMZL	51 (12)	9 (14)		60 (12)
Diss. MZL	68 (16)	10 (15)		78 (16)

BM, bone marrow; ECOG, Eastern Cooperative Oncology Group; PS, performance status; ALC, absolute lymphocyte count; LDH, lactate dehydrogenase; UNL, upper normal limit; B2M, β_2_-microglobulin: ENS: extranodal sites; MZL: marginal zone lymphoma; ENMZL: extranodal marginal zone lymphoma: SMZL: splenic marginal zone lymphoma; NMZL: nodal marginal zone lymphoma; DissMZL: disseminated marginal zone lymphoma.

[n = ]: number of patients where the data was reported, otherwise n = 501.

### Model development

Fourteen covariates were initially identified as potentially relevant for model development that were previously included in other prognostic scores and based on the clinical relevance and easiness of use; in particular, we conducted a cluster analysis using the Hoeffing distance and complete linkage method (as shown in the figure of cluster analysis that we provide below) that helped us to select covariates with clinical values and without meaningful overlap with others. Among these covariates β_2_-microglobulin (B2M) and the longest diameter of largest lymph node (LoDLIN) were subsequently excluded due to high numbers of missing values. Moreover, a strong correlation between B2M and hemoglobin (Hb, Pearson’s rho = −0.42) and between LoDLIN and MZL subtype (Spearman's rho = 0.24) and number of nodal sites (Pearson’s rho = 0.32) was observed suggesting that removing B2M and LoDLIN would not result in loss of prognostic details. Results of the univariable analysis are shown in Table 2. With regards to MZL subtypes, we observed a significant correlation with worse PFS for NMZL and dissMZL compared to SMZL and ENMZL in univariate analysis in the study population and in the larger population of all MZL cases included in the NF10 study (Data Supplement, Supplementary Fig. S4a and b). We then conducted a multivariable analysis on the 456 patients without missing values for the selected 12 covariates, considering the covariates associated with PFS with p < 0.50 in univariable analysis. The final analysis confirmed an independent association with PFS for lactic dehydrogenase (LDH) (< or ≥ upper normal limit, UNL), Hb level (< or ≥12 g/dL), platelet count (< or ≥100 × 10^9^/mmc), absolute lymphocyte count (< or ≥1 × 10^9^/mmc), and MZL subtype (ENMZL and SMZL vs NMZL and dissMZL). Regarding the MZL subtype, its independent prognostic role was confirmed in the unadjusted analysis as well as in the analysis adjusted by potentially confounding covariates such as LDH, Hb and platelet count (Table 2). Further, influential subject did not emerge on the regression coefficients (Supplemental Section D).

**Table 2:** Univariable and final model for progression-free survival on complete cases cohort of marginal zone lymphoma.

Covariate	Status	Univariable		Multivariable model	
		HR (95%CI)	p	HR (95%CI)	p
Age	>70 years	1.71 (1.22–2.39)	0.002		
Sex	F	0.98 (0.70–1.37)	0.923		
Stage	III–IV	2.38 (1.41–4.01)	0.001		
ENS	>1	0.83 (0.54–1.29)	0.415		
Nodal sites	>2	1.48 (1.06–2.08)	0.023		
Symptoms	B	1.38 (0.93–2.04)	0.110		
LoDLIN	>6 cm	1.47 (0.93–2.33)	0.101		
B2M	>ULN	1.84 (1.22–2.78)	0.004		
Timing	After W&W	1.22 (0.66–2.27)	0.528		
LDH	>UNL	2.00 (1.43–2.80)	<0.001	1.60 (1.12–2.30)	0.011
ALC	<1 × 10^9^/L	1.76 (1.20–2.56)	0.003	1.72 (1.17–2.53)	0.006
Hemoglobin	<12 g/dL	1.95 (1.39–2.72)	<0.001	1.61 (1.13–2.30)	0.009
Platelets	<100 × 10^9^/L	2.29 (1.50–3.51)	<0.001	1.86 (1.18–2.92)	0.007
MZL subtype	SMZL/ENMZL	1.00 (reference)		1.00 (reference)	
	NMZL/DissMZL	1.67 (1.19–2.36)	0.003	1.66 (1.17–2.36)	0.004

HR: hazard ratio; F: female; ENS: extranodal sites; W&W: watch and wait; LDH: lactate dehydrogenase; UNL: upper normal limit; ALC: absolute lymphocyte count; MZL: marginal zone lymphoma; SMZL: splenic marginal zone lymphoma; ENMZL; extranodal marginal zone lymphoma; NMZL: nodal marginal zone lymphoma; DissMZL: disseminated marginal zone lymphoma; LoDLIN: longest diameter of the largest involved node; B2M, β_2_-microglobulin.

LR test: Likelihood-ratio test, with reduced model nested in full model.

**Fig. 1:**
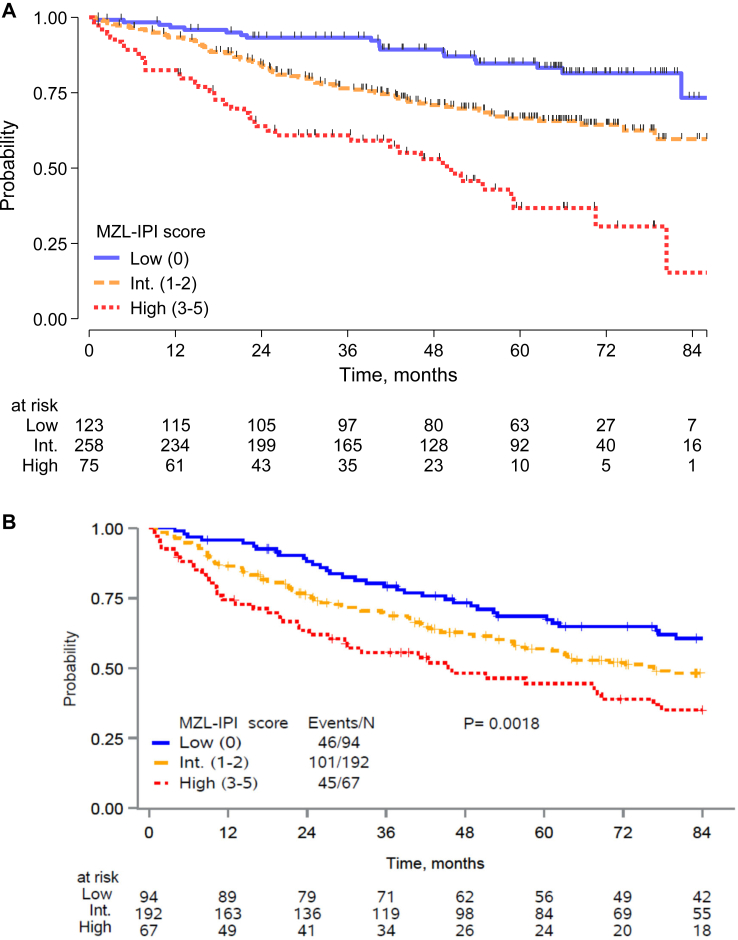
Progression-free survival of patients with marginal zone lymphoma according to MZL-IPI risk category (low, intermediate, high) (training sample) (A) and of patients from US Cohort (the University of Iowa/Mayo Clinic Lymphoma Specialized Program of Research Excellence, SPORE, Molecular Epidemiology Resource, MER, and Sylvester Comprehensive Cancer Center, University of Miami Miller School of Medicine, Miami, FL, USA) (validation sample) (B).

**Fig. 2:**
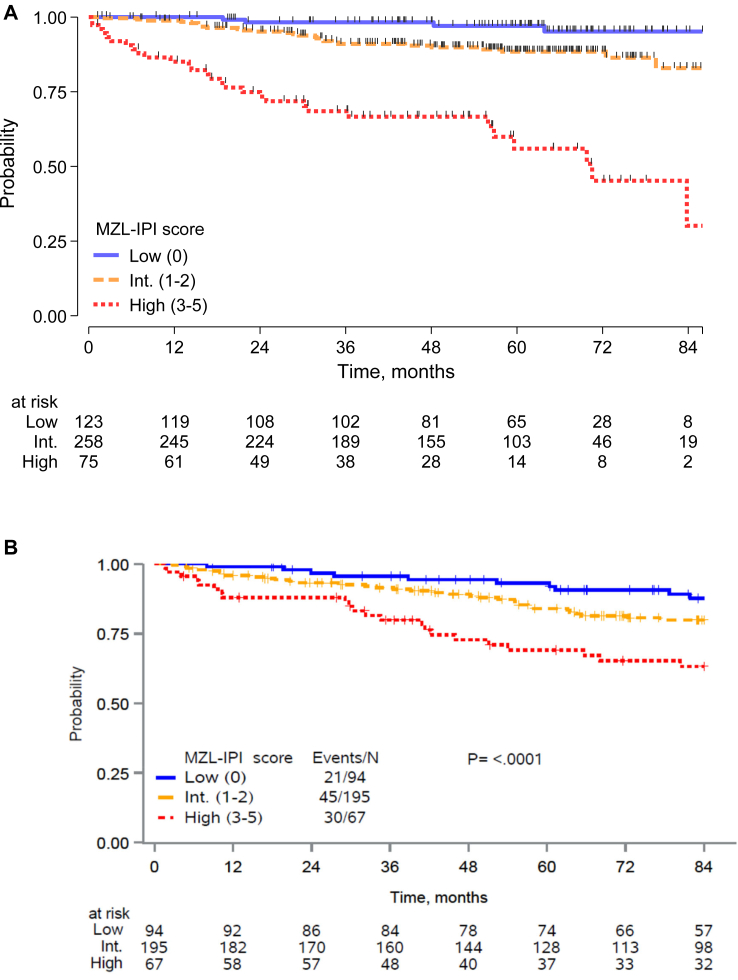
Overall survival of patients with marginal zone lymphoma according to MZL-IPI risk category (low, intermediate, high) (training sample) (A) and of patients from US Cohort (the University of Iowa/Mayo Clinic Lymphoma Specialized Program of Research Excellence, SPORE, Molecular Epidemiology Resource, MER, and Sylvester Comprehensive Cancer Center, University of Miami Miller School of Medicine, Miami, FL, USA) (validation sample) (B).

We considered the z-value from Wald test associated with each final covariate in the multivariable model and we defined a categorical index assigning a score of 1 to each unfavorable feature. A prognostic model was then fit with the 5 factors that categorized patients into 6 groups, with PFS differences by KM and Cox analysis shown in the Data Supplement, Supplementary Fig. S5. Based on the contrast between the log (HRs) by levels of the score from the Cox model (Data Supplement, Supplementary Table S2), we defined the MZL-IPI with the following risk groups: a low-risk group (LRG, 0 factor, 27%), an intermediate-risk group (IRG, 1–2 factors, 57%) and a high-risk group (HRG, 3+ factors, 16%); 5-y PFS was 85% for the LRG, 66% for IRG, and 37% for HRG (Fig. 1a and Table 3a). Compared to the LRG, the IRG (HR = 2.30, 95% CI 1.39–3.80) and HRG (HR = 5.41, 95% CI 3.12–9.38) had inferior PFS. Regarding OS, MZL-IPI identified risk groups associated with a different risk of death between intermediate and low risk (p = 0.020) and high vs intermediate group (p < 0.001) (Fig. 2a; Data Supplement, Supplementary Table S3a).

**Table 3:** MZL-IPI from the final Cox PH model in progression free survival in training (A) and validate sample (B).

A) MZL-IPI in training sample (n = 456)				
Group	N (%) [#fail]	5-yr PFS% (95%CI)	HR (95%CI)	p
Low 0	123 (27) [19]	85 (76–90)	1.00	
Intermediate 1/2	258 (57) [79]	66 (60–72)	2.30 (1.39–3.80)	0.001
High 3/5	75 (16) [40]	37 (23–50)	5.41 (3.13–9.38)	<0.001
High vs Intermediate			2.35 (1.60–3.45)	<0.001

B) MZL-IPI in validation sample (n = 353)				
Group	N (%) [#fail]	5-yr PFS% (95%CI)	HR (95%CI)	p
Low 0	94 (27) [46]	69 (58–77)	1.00	
Intermediate 1/2	192 (54) [101]	57 (49–64)	1.33 (0.94–1.89)	0.108
High 3/5	67 (19) [45]	45 (32–56)	2.08 (1.37–3.14)	<0.001
High vs Intermediate			1.56 (1.10–2.22)	0.013

PFS: progression-free survival.

Any adverse factor was weighted = 1, and the sum of the worst prognostic category ranging from 0 to 5.

We observed 64 early progressions defined by POD24: 6% in low risk, 15% in intermediate risk (OR 2.6, 95% CI 1.13–6.14, p = 0.024) and 34% in high risk (OR 7.67, 95% CI 3.05–19.3, p < 0.001).

### Internal validation of the model

The internal validation showed a good reproducibility of the model, with a slope shrinkage optimism of 0.078. The c-Harrell was 0.657 and after bias correction 0.644. The details are reported in the Data Supplement, Section E.

For sensitivity analysis, the score was recalculated after 10 multiple imputations over the cohort of 501 patients, in which the missing values were replaced in imputed datasets; the MZL-IPI showed a similar performance to the set with only complete values, with HR between intermediate and low group risk of 2.33 (95%CI 1.45 to 3.75) and high vs intermediate of 1.97 (95%CI 1.30 to 3.03), with c-Harrel 0.630. Further, considering the censored patients with follow-up <24 months as competing events (possible masking of failure), the MZL-IPI was similar with estimations in complete cases cohort (details in the Data Supplement, Section F).

Further, the selected variables showed their prognostic role as continuous covariates (details in Data Supplement, Supplementary Table S8).

Finally, no difference in clinical characteristics and outcomes was observed comparing the site of registration (Italy vs other countries) (data not shown).

### External validation of the MZL-IPI

We identified two US cohorts of MZLs (SPORE-MER and Miami) for validation. After harmonizing the data and applying the same inclusion criteria and classification scheme (including dissMZL) used for the NF10 training population, there were 249 eligible patients in the MER and 161 in the Miami cohort. To provide more stable estimates, we elected to pool the cohorts together for a total of 353 MZL patients without missing values for the model covariates. The clinical characteristics of the combined validation cohort as well as of each original cohort are summarized in the Data Supplement, Supplementary Table S4. In the Miami cohort, 37% patients were treated with rituximab monotherapy and 54% with chemotherapy combined with rituximab. In the Mayo dataset, 40% patients were treated with rituximab monotherapy and 46% with chemotherapy combined with rituximab.

In the combined US cohort, after a median follow-up of 77.8 months, the 5-y PFS was 58% (95% CI 52–63). Applying the MZL-IPI to the US cohort, 94 (27%), 192 (54%), and 67 (19%) patients were classified as LRG, IRG, and HRG, respectively. The MZL-IPI was prognostic for PFS with 5-y PFS of 69%, 57% and 45% for low, intermediate, and high risk (log-rank test p = 0.0018; c-Harrell = 0.578) (Fig. 1b and Table 3b). Details about prognostic performance of MZL-IPI for PFS within the two distinct validation cohorts (SPORE-MER and Miami) are provided in the Data Supplement (Supplementary Figs. S6 and S7 and Tables S5 and S6); the prognostic model performed differently in the 2 validation cohorts with the main difference being represented by a different PFS of the intermediate risk group. Conversely, both the low and the high-risk groups were well separated in both series. It is also important that in both series the prognostic order of the risk groups defined by the score was maintained.

When tested for OS, the ability to identify groups at different risk of death was confirmed, with 5-yr OS for low, intermediate, and high risk of 93%, 84% and 69%, respectively (log-rank test p < 0.001 and c-Harrell 0.581, Fig. 2b; Data Supplement, Supplementary Table S3b).

## Discussion

In this study, we developed and externally validated the MZL-IPI as a new prognostic score for all patients with MZL who are being considered for systemic treatment. The MZL-IPI is based on the independent prognostic role of five easy-to-assess and commonly available variables and has been shown to be accurate in predicting both PFS and OS in both the training and the validation sets.

Among the independent prognostic factors included in the final model, anemia, thrombocytopenia and elevated LDH have been frequently reported as adverse prognostic factors in MZL.^12^, ^13^, ^14^ In addition, in the MZL-IPI, we include MZL subtype as one of the independent factors for survival since both nodal and disseminated MZL were independently associated with unfavorable outcomes. DissMZL were identified as cases of widespread disease without clear primary splenic, nodal or extranodal origin. The existence of disseminated cases^19^ is well recognized; in addition extrahilar adenopathy in SMZL and stage III-IV have been reported as adverse prognostic factors in SMZL and ENMZL, respectively.^13^^,^^14^^,^^20^ The use of disseminated presentation of MZL (dissMZL) in the prognostication of MZL is aimed to improve the general approach to MZL as a group both for clinical research and for therapeutic purposes.

Based on our results, MZL-IPI represents a step forward in the prognostic assessment of MZL. The MZL-IPI is prospectively constructed using MZL patients with all the clinical subtypes and integrates histological, clinical and laboratory parameters specifically selected and validated in the entire spectrum of MZL. Importantly, age was not included in the MZL-IPI, which represents a significant conceptual difference in comparison to other models and may help to better manage an indolent disease such as MZL in the chemo-free era without age limitations to systemic treatment. Finally, compared to other models the MZL-IPI is much easier to apply as stage and calculation of nodal sites is not required.

The MZL-IPI has been developed using PFS as primary study endpoint but was also prognostic for OS further enhancing its value. In addition to both PFS and OS, other endpoints have been recently suggested as surrogate factors for outcome. In particular, we demonstrated along with other groups that experiencing early progression from diagnosis or treatment initiation was related to inferior survival in MZL^16^^,^^29^, ^30^, ^31^, ^32^ and greater risk of death due to lymphoma.^33^ Achievement of CR to initial therapy was demonstrated as the most predictive variable for PFS and OS.^28^ Recently, time to complete response within 24 months (TTCR24) and complete response at 24 months (CR24) were identified as good surrogate markers of PFS.^34^ We elected to focus on PFS as it remains a more conventional and accepted primary outcome measure in clinical trials. While MZL-IPI was not developed to predict early events, 34% of patients in high MZL-IPI-risk category experienced POD24; a full validation of MZL-IPI considering alternative endpoints to PFS is warranted.

The MZL-IPI has been validated in an independent US cohort of MZL derived from the pooling of the MER (monocentric) and Miami series (multicentric). Some differences between the two populations were observed: the US cohort comprised of younger patients, with longer median follow-up and inferior outcomes in comparison to the NF10 cohort. Of note, MZL-IPI was validated but with non-significant pairwise comparisons among groups in the separated cohorts from US (MER and Miami) (Data Supplement): in particular, the main difference being represented by a different PSF of the intermediate risk group. Conversely, both the low and the high risk groups were well separated in both series. Also important in both series the prognostic order of the risk groups defined by the score was maintained. This is likely to be the result of patient selection and of the small size of the separate cohorts but may also reflect a dependency of the model on differences in definition of symptomatic patients and local therapeutic approaches adopted, as preferred treatment choices for symptomatic patients. While these differences need to be acknowledged, the validation of the MZL-IPI in the pooled two US cohorts is a robust approach for confirmation as it better reproduces the multicentric approach used to collect cases for the training NF10 series.

Despite the relatively high number of our prospectively collected cases and the robustness of the results, we acknowledge some limitations in our study. Forty-five (9%) patients were excluded from the model definition because of missing data and although the main covariates related to MZL have been collected, we cannot exclude that other variables may have a role in defining MZL outcomes.

Moreover, in the real-life setting used to recruit patients for both the training and the validation sets, treatment choice was left to the physician's discretion with possible differences across all centers that could affect survival. Further, details on treatment modifications, which can contribute to therapy success, are sometimes incomplete or even lacking altogether. Finally, a formal histological revision has not been performed and applicability of MZL in relapsed/refractory patients treated with chemo-free approach (e.g., Bruton tyrosine kinase (BTK) inhibitors or rituximab plus lenalidomide) is not demonstrated. While these limitations should be acknowledged, we believe that, overall, both the training and the validation sets represent comparable real-life populations managed in the modern treatment era.

The MZL-IPI is the first validated prognostic index designed for the whole spectrum of newly diagnosed symptomatic MZLs that integrates histology and disease dissemination evaluation into a single model contributing to improved patient assessment. The MZL-IPI is a reproducible tool that can be easily calculated, and that mainly identifies a high-risk group of patients with MZL associated with poor outcomes, which identifies a patient population with an unmet need for future investigations. The MZL-IPI is not intended to be used as a predictive score to support clinical and therapeutic decisions for the individual patient, but it represents an important tool to aid clinical development and to allow improvement in clinical trial designs, results interpretation, and cross trial comparisons.

## Contributors

**Conception and design:** Luca Arcaini, Stefano Luminari, Luigi Marcheselli.

**Provision of study materials or patients:** Luca Arcaini, Côme Bommier, Juan Pablo Alderuccio, Michele Merli, Nicole Fabbri, Maria Elena Nizzoli, Matthew J. Maurer, Vittoria Tarantino, Simone Ferrero, Sara Rattotti, Annalisa Talami, Roberta Murru, Arushi Khurana, Raphael Mwangi, Marina Deodato, Emanuele Cencini, Francesca Re, Carlo Visco, Andrew L. Feldman, Brian K. Link, Marcia Torresan Delamain, Michele Spina, Ombretta Annibali, Alessandro Pulsoni, Andrès J. M. Ferreri, Caterina Cecilia Stelitano, Elsa Pennese, Thomas M. Habermann, Luigi Marcheselli, Marco Paulli, Izidore S. Lossos, James R. Cerhan, Stefano Luminari.

**Collection and assembly of data:** All authors.

**Data analysis and interpretation:** Luca Arcaini, Stefano Luminari, Luigi Marcheselli, Marco Paulli, Côme Bommier, Juan Alderuccio, Matthew J. Maurer, Sunwoo Han, Isildinha M. Reis, Izidore S. Lossos, James R. Cerhan.

**Manuscript writing:** All authors.

**Final approval of manuscript:** All authors.

**Accountable for all aspects of the work:** All authors.

## Data sharing statement

Qualified researchers may contact the FIL board at segreteriadirezione@filinf.it to share individual-level patients’ clinical data analyzed for this manuscript (for the avoidance of doubt, no identifiable data, such as name, address, hospital name, date of birth, or any other identifying data, will be shared and should not be requested). For each data sharing request, it is essential that a *proforma* (available on request) is completed that describes the general purpose, specific aims, data items requested, analysis plan and acknowledgment of the trial management team. Requests will be reviewed based on scientific merit and ethical principles. Requestors who are granted access to the data will be required to complete a data sharing agreement that will be signed by the requester and FIL. In compliance with the domestic ethics guideline and applicable legislation, individual deidentified patients’ data underlying the results reported in this Article (including study protocol, statistical analysis plan and data coding) can be shared until 5 years after the publication of the present article.

## Declaration of interests

LA: Grants or contracts from any entity: My First AIRC grant n. 11,415 2012–2014; Investigator Grant AIRC (2018–2022); Honoraria: EUSA Pharma, Novartis. Participation on a Data Safety Monitoring Board or Advisory Board Roche, Janssen-Cilag, Verastem, Incyte, EUSA Pharma, Celgene/Bristol Myers Squibb, Kite/Gilead, ADC Therapeutics, Novartis; Support for attending meetings and/or travel: Roche.

CB: Grants or contracts from any entity: INSERM, AvieSan ITMO Cancer, LYSA/ELI: Bertrand Coiffier Prize Institut Servier; Consulting fees: Currety; Support for attending meetings and/or travel: Mayo Clinic.

JPA: Grants or contracts from any entity: Lymphoma Research Foundation, US Department of Defense; Payment or honoraria for lectures, presentations, speakers bureaus, manuscript writing or educational events: ADC Therapeutics, Regeneron, Genentech.

MM: Support for attending meetings and/or travel: Janssen.

MJM: Grants or contracts from any entity: BMS, Roche, GenMab; Consulting fees: BMS; Participation on a Data Safety Monitoring Board or Advisory Board: AstraZeneca.

CV: Payment or honoraria for lectures, presentations, speakers bureaus, manuscript writing or educational events: Janssen, Lilly, Novartis, Gilead, Takeda, Kyowa-Kirin, Roche, Astra Zeneca, Beigene, Gentili.

BKL: Grants or contracts from any entity: Roche/Genentech, Seattle Genetics, Genmab, AstraZeneca.

OA: Payment or honoraria for lectures, presentations, speakers bureaus, manuscript writing or educational events: Roche, Janssen, Beigene, Ely Lilli, Amgen, Sanofi.

AP: Payment or honoraria for lectures, presentations, speakers bureaus, manuscript writing or educational events: Roche, Msd, Pfizer, Sandoz, Takeda, Gilead, Bms, Janssen, Beigene; Participation on a Data Safety Monitoring Board or Advisory Board: Takeda, Roche.

TMH: All support for the present manuscript (e.g., funding, provision of study materials, medical writing, article processing charges, etc.): Lymphoma SPORE NCI CA 97274; Participation on a Data Safety Monitoring Board or Advisory Board: Seagen, Eli Lilly & Co.

LM: Other financial or non-financial interests: Scientific consultant for Sandoz spa, 2022–2023, free of fee.

JRC: All support for the present manuscript (e.g., funding, provision of study materials, medical writing, article processing charges, etc.): National Cancer Institute, Grants P50 CA97274 and U01 CA195568 (to Mayo); Grants or contracts from any entity: BMS, Genentech, and Genmab; Participation on a Data Safety Monitoring Board or Advisory Board and SMC member: Protagonist Therapeutics.

SL: Payment or honoraria for lectures, presentations, speakers bureaus, manuscript writing or educational events: Roche, Novartis, Incyte, BMS, Kite, Regeneron, Abbvie, Genmab, Sobi, Beigene; Support for attending meetings and/or travel: Roche, Beigene, Regeneron.

NF, MEN, VT, SF, SR, AT, RM, AK, RM, MD, EC, FR, ALF, MDT, MS, AJMF, CS, EP, SH, IMR, ISL: no COI.
