# Supplementary material for: Marginal zone lymphoma international prognostic index: a unifying prognostic index for marginal zone lymphomas requiring systemic treatment
Source: eClinicalMedicine. 2024 Apr 11;72:102592. doi: 10.1016/j.eclinm.2024.102592 (PMC11019091; doi:10.1016/j.eclinm.2024.102592)
Supplement: Supplementary data, Figs. S1–S7, and Tables S1–S8 [file mmc1.docx]

**Data Supplement to manuscript**

**“Marginal Zone Lymphoma International Prognostic Index: a unifying prognostic index for marginal zone lymphomas requiring systemic treatment”** by L. Arcaini, C. Bommier, J. P. Alderuccio, M. Merli, N. Fabbri, M. E. Nizzoli, M. J. Maurer, V. Tarantino, S. Ferrero, S. Rattotti, A. Talami, R. Murru, A. Khurana, R. Mwangi, M. Deodato, E. Cencini, F. Re, C. Visco, A. L. Feldman, B. K. Link, M. Torresan Delamain, M. Spina, O. Annibali, A. Pulsoni, A. J. M. Ferreri, C. Stelitano, E. Pennese, T. M. Habermann, L. Marcheselli, S. Han, I. M. Reis, M. Paulli, I. S. Lossos, J. R. Cerhan and S. Luminari

**Contents**

**Supplementary data and statistical methods**

**Section A) List of centres……………………………………………...............................................4**

**Section B) NF10 study inclusion criteria and details of MZL-IPI analysis .............................5**

**Section C) External validation cohorts…………………………………………….........................6**

**Section D) Diagnosis of final Cox proportional hazard regression model……………….……...8**

**Section E) Internal validation……………………………………………………………………...9**

**Section F) Sensitivity analyses…………………………………………………………………….10**

**Supplementary Tables**

**Table S1.** Rules adopted to define subtypes of marginal zone lymphomas…………………..........13

**Table S2.** Number of patients, events, 5-year progression-free survival, hazard ratio and log-rank test p-values according to the 6 (0 to 5) factors of the prognostic score……………………………14

**Table S3.** Prognostic role of MZL-IPI on overall survival in the training and in the validation sets………………………………………………………………………………………………….15

**Table S4.** Clinical characteristics of the external validation (the University of Iowa/Mayo Clinic Lymphoma Specialized Program of Research Excellence, SPORE, Molecular Epidemiology Resource, MER, and Sylvester Comprehensive Cancer Center, University of Miami Miller School of Medicine, Miami, FL, USA) data………………………………………………………………..16

**Table S5.** Progression-free survival by MZL-IPI of patients from the University of Iowa/Mayo Clinic Lymphoma Specialized Program of Research Excellence, SPORE, Molecular Epidemiology Resource, MER……………………………………………………………………………………..17

**Table S6.** Progression-free survival by MZL-IPI of patients from the Sylvester Comprehensive Cancer Center, University of Miami Miller School of Medicine, Miami, FL, USA……………….18

**Table S7.** Comparison of Hazard rations among all patients, patients treated with immunochemotherapy and patients treated with immunochemotherapy and Rituximab alone……19

**Table S8.** Cox PH model with covariates in continuous form……………………………………….20

**Supplementary Figures**

**Figure S1.** Flow-chart of patients with indolent non-follicular lymphoma enrolled in NF10 study of Fondazione Italiana Linfomi………………………………………………………………………..21

**Figure S2. a)** Progression-free survival for all 501 MZL treated patients; **b)** Progression-free survival for patients treated immediately after diagnosis or after progression from watct and wait………………………………………………………………………………………………….22

**Figure S3.**  Overall survival for all 501 MZL patients……………………………………………..23

**Figure S4. a)** Progression-free survival by subtype of marginal zone lymphoma in all 790 patients; **b)** Progression-free survival by subtype of marginal zone lymphoma in the 501 treated patients considered for model definition……………………………………………………………………..24

**Figure S5.** Progression-free survival stratified by the 6 (0 to 5) factors of the prognostic score……..25

**Figure S6.** Progression-free survival of patients with marginal zone lymphoma according to MZL-IPI risk category (low, intermediate, high) of patients from the University of Iowa/Mayo Clinic Lymphoma Specialized Program of Research Excellence, SPORE, Molecular Epidemiology Resource, MER……………………………………………………………………………………...26

**Figure S7.** Progression-free survival of patients with marginal zone lymphoma according to MZL-IPI risk category (low, intermediate, high) of patients from Sylvester Comprehensive Cancer Center, University of Miami (UM) Miller School of Medicine, Miami, FL, USA…………………………27

**Supplementary data and statistical methods**

**Section A) List of centres**

- Division of Hematology, Fondazione IRCCS Policlinico San Matteo, Pavia, Italy
- Division of Hematology, Azienda Unità Sanitaria Locale – IRCCS, Reggio Emilia, Italy
- Division of Hematology and Stem Cell Transplantation, Ospedale A. Businco, Cagliari, Italy
- Division of Hematology, University Hospital Ospedale di Circolo e Fondazione Macchi-ASST Sette Laghi, University of Insubria, Varese, Italy
- Division of Hematology, University of Torino, Department of Molecular Biotechnologies and Health Sciences, University of Torino, and AOU “Città della Salute e della Scienza di Torino”, Presidio “Le Molinette”, Torino, Italy
- Division of Hematology, Niguarda Cancer Center, ASST Grande Ospedale Metropolitano Niguarda, Milano, Italy
- Division of Medical Oncology, Centro di Riferimento Oncologico, Aviano National Cancer Institute Aviano, Aviano, Italy
- Center of Hematology and Hemotherapy, Department of Internal Medicine, Faculty of Medicine, State University of Campinas, Campinas, Brazil
- Division of Hematology, Azienda Ospedaliero-Universitaria di Parma, Parma, Italy
- Division of Hematology, Azienda Ospedaliera Universitaria Senese and University of Siena, Siena, Italy
- Division of Hematology, Ospedale San Bortolo, Vicenza, Italy
- Oncology Unit 1, Veneto Institute of Oncology IOV-IRCCS, Padua, Italy
- Division of Hematology, Sapienza University – Polo Pontino, Department of Translational and Precision Medicine, S.M. Goretti Hospital, Latina, Italy
- Division of Hematology, Stem Cell Transplantation, University Campus Bio-Medico, Rome, Italy
- Lymphoma Unit, Department of Onco-Hematology, IRCCS San Raffaele Scientific Institute, Milano, Italy
- Division of Hematology, Grande Ospedale Metropolitano, Bianchi Melacrino Morelli, Reggio Calabria, Reggio Calabria, Italy
- Lymphoma Unit, Department of Hematology, Ospedale Spirito Santo, Pescara, Italy
- Division of Hematology, AOU “Città della Salute e della Scienza di Torino”, Presidio “Le Molinette”, Torino, Italy.
- Division of Hamtoloy, Presidio Ospedaliero Madonna delle Grazie , Matera , Italy
- Unit of Medical oncology, Ospedale San Paolo,, ASST San Paolo e Carlo, Milano, Italy
- Division of Hematology, Department of Translational Medicine, University of Eastern Piedmont, Novara, Italy
- Division of Hematology, Presidio ospedaliero "A. TORTORA", Pagani, Italy
- Universidade Federal do Rio de Janeiro, Brazil
- Irmandade da Santa Casa de Misericórdia de São Paulo, Brazil
- Hematology Unit, Department of Oncology and Hematology, Azienda-Ospedaliero Universitaria (AOU) of Modena, Modena, Italy
- Division of Hematology, San Gerardo Hospital, Monza
- Division of Hematology, Azienda Ospedaliera Papardo. Messina, Italy
- Oncology Unit, Azienda Unità Sanitaria Locale Modena, Area Sud Sede di Sassuolo, Sassuolo, Italy
- Humanitas Cancer Center, IRCCS Humanitas Research Hospital, Rozzano, Milano, Italy
- Division of Hematology, AO San Carlo Borromeo, ASST Santi Paolo e Carlo, Milano, Italy
- Division of Hematology, Fondazione IRCCS Ca' Granda Ospedale Maggiore Policlinico, Milano, Italy
- Haematology Unit, Instituto Portugues de Oncologia de Lisboa Francisco Gentil, Lisbon, Portugal
- Division of Hematology, Azienda Ospedalaliera S. Maria di Terni, Terni, Italy
- Unit of Hematology and Stem Cell Transplantation, AOU Policlinico Consorziale, Bari, Italy
- Division of Internal Medicine I, Division of Oncology, Medical University Vienna, Vienna, Austria
- Kiev National Cancer Institute, Kiev, Ukraine
- Division of Hematology, IRCCS CROB of Rionero in Vulture, Rionero in Vulture, Italy
- Division of Hematology, Department of Translational and Precision Medicine, "Sapienza" University, Rome, Italy
- Division of Hematology, Garibaldi Nesima Hospital, Catania, Italy
- Division of Hematology, Ospedale Valduce, Como, Italy
- Division of Hematology Unit, Department of Hemato-Oncology, Ospedale Annunziata, Cosenza, Italy
- Hemato-Oncology Department, DMU DHI, Hôpital Saint Louis, Paris, France
- Division of Hematology, Hospital Vito Fazzi, Lecce, Italy
- Division of Hematology, Azienda Ospedaliera Papa Giovanni XXIII, Bergamo, Italy
- Division of Hematology, AOU Pisana, Pisa, Italy
- Division of Hematology, Ospedale "S.G. Moscati", Taranto, Italy

**Section B) NF10 study inclusion criteria and details of MZL-IPI analysis**

Inclusion criteria of NF10 study required a diagnosis of SMZL, ENMZL, NMZL, lymphoplasmacytic lymphoma, small lymphocytic lymphoma, and CD5-negative indolent lymphoma. A tissue biopsy of a nodal or extranodal lesion or a bone marrow biopsy consistent with histological diagnosis of MZL, assessed by a local hemopathologist, was mandatory. Moreover, if available, peripheral and/or bone marrow flow cytometry analysis was collected.

Patients with histologic features of MZL who had a concomitant involvement of the marrow and/or spleen and/or lymph nodes and/or extranodal sites were categorized as dissMZL. An operational approach to MZL grouping and to define dissMZL is provided in the Data Supplement, Table S1. Clinical characteristics and radiologic assessment were recorded at time of diagnosis. Laboratory reports included data on serology for human immunodeficiency virus, hepatitis B and C virus status, lactate dehydrogenase level (LDH), β_2_-microglobulin (B2M), albumin, absolute lymphocyte count (ALC) and platelets, with a focus on factors from commonly used prognostic score for MZL: MALT-IPI for ENMZL^1^, SMZL scores^2,3^, and Follicular Lymphoma International Prognostic Index (FLIPI) for nodal and dissMZL.^4^ Patients were managed according to local institutional guidelines. Initial treatment decision following diagnosis was collected with details about planned and administered treatment. Response assessment was done according to NF10 protocol that required the reassessment of all sites of disease identified at baseline with CT scan, bone marrow biopsy and additional organ specific exams when appropriate. Follow up procedures were also managed according to the NF10 protocol with a minimum requirement of one assessment every year. Radiologic follow up examination were required as per protocol.

1. Thieblemont C, Cascione L, Conconi A, et al. A MALT lymphoma prognostic index. *Blood*. 2017;130(12). doi:10.1182/blood-2017-03-771915

2. Arcaini L, Lazzarino M, Colombo N, et al. Splenic marginal zone lymphoma: A prognostic model for clinical use. *Blood*. 2006. doi:10.1182/blood-2005-11-4659

3. Montalban C, Abraira V, Arcaini L, et al. Simplification of risk stratification for splenic marginal zone lymphoma: A point-based score for practical use. *Leuk Lymphoma*. 2014;55(4). doi:10.3109/10428194.2013.818143

4. Arcaini L, Paulli M, Burcheri S, et al. Primary nodal marginal zone B-cell lymphoma: Clinical features and prognostic assessment of a rare disease. *Br J Haematol*. 2007. doi:10.1111/j.1365-2141.2006.06437.x

**Section C) External validation cohorts**

**Molecular Epidemiology Resource:** The University of Iowa/Mayo Clinic Lymphoma Specialized Program of Research Excellence (SPORE) Molecular Epidemiology Resources (MER) is a prospective cohort study that offered enrollment to consecutive, newly diagnosed patients at the University of Iowa and Mayo Clinic Rochester from 2002-2015 [1]. Patients provided informed consent for participation in the MER and the use of their data for future research. Demographic, clinical, and pathologic data were abstracted using a standard protocol. Pathologic confirmation was conducted centrally by MER-affiliated expert hematopathologists using current World Health Organization criteria at the time of diagnosis. All participants were managed per treating physician and were prospectively contacted every 6 months for the first 3 years and then annually thereafter to identify disease progression, retreatment, transformation, and death, all of which were validated against medical records. This analysis is based on participants with MZL who were treated with systemic chemotherapy or immunotherapy at diagnosis, and follow-up was through 2022.

**University of Miami:** University of Miami MZL cohort used for this analysis is based on patients with MZL identified via the Florida Cancer Registry that were diagnosed/treated at Sylvester Comprehensive Cancer Center, Bascom Palmer Eye Institute, University of Miami Hospital, and Jackson Memorial Hospital from June 1995 to April 2022. The institutional review board of the University of Miami approved this study, which followed the tenets of the Declaration of Helsinki. The cohort is updated annually and the last follow up for patients included in this study was on 7/23/2023.

All diagnostic specimens were reviewed by expert hematopathologists working at University of Miami Department of Pathology using the morphologic and immunophenotypic diagnosis of MZL defined by the WHO classification. Medical records are reviewed to obtain patient demographics, laboratory findings, staging, treatments, dates of diagnosis, relapse, transformation, and death or last follow-up. As of 7/23/2023, the cohort consisted of 715 patients with a pathologically confirmed diagnosis and available key clinical data. Staging evaluation was not standardized during the study interval but included a complete physical examination; hematologic and chemical survey with lactate dehydrogenase (LDH); computed tomography scan of the chest, abdomen, and pelvis; and magnetic resonance imaging, if indicated. Positron emission tomography/computed tomography was not routinely performed at diagnosis until 2019, when the UM lymphoma program has consistently incorporated PET/CT in the staging workup of patients with EMZL independently of concerns for high-grade transformation. The decision to perform a staging bone marrow biopsy and β2-microglobulin was at the discretion of the treating oncologist. The patients are managed at the discretion of treating physicians, and many are presented and discussed at the Lymphoma board meeting weekly. This analysis is based on MZL who were treated with systemic chemotherapy or immunotherapy at diagnosis.

**References**

1. Cerhan et al. Cohort Profile: The Lymphoma Specialized Program of Research Excellence (SPORE) Molecular Epidemiology Resource (MER) Cohort Study. Int J Epidemiol 2017 Dec 1;46(6):1753-1754.

**Section D) Diagnosis of final Cox proportional hazard regression model**

- Analysis of residuals and influential subjects on the estimators of all coefficients (likelihood displacement or Cook’s distance)

o: censored, x: events.

Influential subjects did not emerge on the regression coefficients, and the martingale residual showed an acceptable pattern

**- Test of proportional-hazards assumption** (logarithmic time function): Chi2 8.09, p=0.151

**Section E): Internal validation**

| **Internal Validation model** | |  |  |  |
| --- | --- | --- | --- | --- |
| C-index Harrell | Reference: 0.657 (0.610-0.704). Bias corrected: 0.644 | | | |
| Slope Shrinkage | Optimism: 0.078, slope: 0.922 | | | |

C-index Harrell: bias corrected after 1000 bootstrap resamples.

Slope shrinkage: shrinkage coefficient quantifying the overfitting after 1000 bootstrap resamples.

Overfitting acceptable with shrinkage >0.90.

**- Internal calibration of Cox PH regression model**

Performed over 250 bootstrap resamples. About 100 cases per group. The Y axis represents the observed probability of overall 5-year survival from Kaplan-Meier (KM) estimates; the X axis represents the predicted 5-year probability of progression free survival.

**Section F) Sensitivity analyses**

Given that some values were missing and that confounding factors may not have been measured, we performed a sensitivity analysis to check the stability of the Cox PH model and MZL-PI score. The sensitivity analysis was evaluated by two approaches. After multiple imputation (MI) by means of chained equations (10 imputed dataset) and applying the Rubin’s rules to combine estimate coefficients across the M imputed datasets [1]. Continuous covarites were imputed with linear regression [log(ratio LDH/LDHmax), log(B2M/B2Mmax), log(Lymphocytes count), Hemoglobin, log(Platelet count)] and binary covariates with logit regression (stage III/IIV, extranodal sites>1, Nodal size> 6 cm). For categorical covariates, in presence of perfect prediction was performed augmented regression. The covariates age, sex, histology and cumulative risk of progression (according to Nelson-Aalen estimator) were completed in the database. In the imputed datasets the continuous covariates were dichotomized as in the original model. The censored patients with follow-up <24 months have been treated a) as competing events (possible masking of failure) and fitting the model by Fine-Gray approach [2], b) as alive patients with follow-up of 96 months and c) as failures at time of censoring.

**References**

1) White IR, Royston P, Wood AM. Multiple Imputation Using Chained Equations: Issues and Guidance for Practice. Stat Med 2011; 30:377-399

2) Fine JP, Gray RJ. A proportional hazards model for the subdistribution of a competing risk. Journal of the American Statistical Association. 1999; 94: 496-509

**Sensitivity Analysis: Multiple Imputation (m=10, sample size n=501).**

| **Covariate** | **Status** | **HR (95%CI)** | **RVI** | **FMI** | **%iSE** |
| --- | --- | --- | --- | --- | --- |
| LDH | >UNL | 1.50 (1.04-2.16) | 0.059 | 0.056 | 2.89 |
| ALC | <1 x 10^9^/L | 1.46 (0.96-2.21) | 0.044 | 0.043 | 2.20 |
| Hemoglobin | <12 g/dL | 1.59 (1.13-2.25) | 0.012 | 0.012 | 0.61 |
| Platelets | <100 x 10^9^/L | 1.73 (1.11-2.70) | 0.030 | 0.029 | 1.48 |
| Subtype of MZL | NMZL/DissMZL | 1.49 (1.06-2.09) | 0.007 | 0.007 | 0.36 |
| **Score** |  | **HR (95%CI)** | **RVI** | **FMI** | **%iSE** |
|  | Low (0) | 1.00 |  |  |  |
|  | Intermediate (1-2) | 2.33 (1.45-3.75) | 0.061 | 0.058 | 3.00 |
|  | High (3-5) | 4.59 (2.66-7.92) | 0.073 | 0.069 | 3.61 |
|  | High vs Intermediate | 1.97 (1.30-3.03) |  |  |  |

MI: multiple imputation; m= number of imputed datasets: Cox model over 501 cases

RVI: average relative increase variance between missing; FMI: fraction of missing information; %iSE= percentage increase of standard error

HR: Hazard Ratio; CI: Confidence Interval; LDH: lactate dehydrogenase; ALC: absolute lymphocytes count

5-yr PFS in imputed samples (m=10, n=501) by levels of proposed score:

Dotted lines: 5-yr PFS by score observed in compete cases dataset (n=456).

**Sensitivity Analysis: treating the censoring in PFS within 24 months of follow-up as a) competing event, b) alive with follow-up of 96 months; c) fails**

On 456 patients 28 were censored within 24 months of follow-up in PFS (6%)

|  | **Original** | **Competing Risk** | **As Alive** | **As Events** |
| --- | --- | --- | --- | --- |
| **Covariate** | **HR (95%CI)** | | | |
| LDH>UNL | 1.60 (1.12-2.30) | 1.56 (1.09-2.23) | 1.53 (1.07-2.19) | 1.52 (1.10-2.11) |
| ALC<1 x 10^9^/L | 1.72 (1.17-2.53) | 1.65 (1.12-2.43) | 1.69 (1.15-2.49) | 1.48 (1.04-2.11) |
| Hemoglobin <12 g/dL | 1.61 (1.13-2.30) | 1.58 (1.11-2.57) | 1.56 (1.09-2.22) | 1.54 (1.12-2.11) |
| Platelets<100 x 10^9^/L | 1.86 (1.18-2.92) | 1.79 (1.14-2.82) | 1.63 (1.04-2.56) | 1.80 (1.20-2.70) |
| NMZL/DissMZL | 1.66 (1.17-2.36) | 1.74 (1.23-2.46) | 1.68 (1.18-2.39) | 1.42 (1.03-1.96) |
| **Score** | **HR (95%CI)** | | | |
| Low (0) | 1.00 | 1.00 | 1.00 | 1.00 |
| Intermediate (1-2) | 2.30 (1.39-3.80) | 2.26 (1.38-3.71) | 2.27 (1.37-3.75) | 1.83 (1.21-2.78) |
| High (3-5) | 5.41 (3.13-9.38) | 5.20 (3.04-3.71) | 5.01 (2.90-8.67) | 3.88 (2.43-6.20) |
| High vs Intermediate | 2.35 (1.60-3.45) | 2.29 (1.57-3.35) | 2.21 (1.51-3.23) | 2.12 (1.49-3.01) |

The covariate and score MZL-IPI retain their prognostic role

**Supplementary Tables**

**Table S1.** Rules adopted to define subtypes of marginal zone lymphomas

|  | **Extranodal sites other than bone marrow** | **Spleen*** | **Lymph node**** | **Bone marrow** | **Peripheral blood***** |
| --- | --- | --- | --- | --- | --- |
| **ENMZL** | Required (single or multiple sites) | No | Only regional LN allowed | Allowed | No |
| **SMZL** | No | Required | Only splenic hilrar LN | Allowed | Allowed |
| **NMZL** | No | No | Required | Allowed | No |
| **DissMZL** | Allowed | Allowed | Allowed | Allowed | Allowed |

Legend:

*Splenic involvement defined in case of splenic biopsy and/or nodular involvement and/or splenic enlargement (>13 cm in max diameter)

** as per Cheson 2007 criteria

*** clonal B-lymphocytes with features (cytology and/or FLowCy) consistent with MZL. Cases with only PB involvement but without splenic or nodal or LN involvement were excluded.

BM: bone marrow; LN: lymph node.

**Table S2.** Number of patients, events, 5-year progression-free survival, hazard ratio and log-rank test p-values according to the 6 (0 to 5) factors of the prognostic score (any adverse factor was weighted = 1, and the sum of the worst prognostic category ranging from 0 to 59)

| **Score level (n=456)** | **N (%)** | **Fails, n (%)** | **5-yr PFS% (95%CI)** | **HR (95%CI)** | **P** |
| --- | --- | --- | --- | --- | --- |
| 0 | 123 (27) | 19 (15) | 85 (76-90) | 1.00 |  |
| 1 | 151 (33) | 41 (27) | 72 (63-79) | 2.00 (1.16-3.44) | 0.013 |
| 2 | 107 (24) | 38 (36) | 58 (47-68) | 2.78 (1.60-4.82) | <0.001 |
| 3 | 61 (13) | 31 (51) | 41 (26-55) | 4.78 (2.69-8.46) | <0.001 |
| 4 | 11 (2) | 6 (55) | NA | 8.40 (3.33-21.2) | <0.001 |
| 5 | 3 (1) | 3 (100) | NA | 21.4 (6.23-73.4) | <0.001 |
| **Coefficient contrast on previous level of prognostic score** | | | |  |  |
| **Score level** | **df** | **Chi2** | **p-value** |  |  |
| 1 vs 0 | 1 | 6.18 | 0.013 |  |  |
| 2 vs 1 | 1 | 2.16 | 0.142 |  |  |
| 3 vs 2 | 1 | 5.00 | 0.025 |  |  |
| 4 vs 3 | 1 | 1.59 | 0.207 |  |  |
| 5 vs 4 | 1 | 1.74 | 0.188 |  |  |

**Table S3.** Prognostic role of MZL-IPI on overall survival in the training (A) and in the validation sets (B)

| **A) MZL-IPI in training set (n=456)** | | | | |
| --- | --- | --- | --- | --- |
| **Group** | **N (%) [#fail]** | **5-yr OS% (95%CI)** | **HR (95%CI)** | **P** |
| Low (0) | 123 (27) [4] | 97 (91-99) | 1.00 |  |
| Intermediate (1-2) | 258 (57) [27] | 88 (83-92) | 3.46 (1.21-9.89) | 0.020 |
| High (3-5) | 75 (16) [29] | 56 (40-69) | 17.2 (6.04-49.1) | <0.001 |
| High vs Intermediate |  |  | 4.97 (2.94-8.42) | <0.001 |
| **B) MZL-IPI in validation set (n=353)** | | | | |
| **Group** | **N (%) [#fail]** | **5-yr OS% (95%CI)** | **HR (95%CI)** | **P** |
| Low (0) | 94 (27) [21] | 93 (85-97) | 1.00 |  |
| Intermediate (1-2) | 192 (54) [45] | 84 (78-89) | 1.34 (0.78-2.29) | 0.286 |
| High (3-5) | 67 (19) [30] | 69 (56-79) | 3.00 (1.68-5.33) | <0.001 |
| High vs Intermediate |  |  | 2.24 (1.41-3.55) | <0.001 |

**Table S4.** Clinical characteristics of the external validation (the University of Iowa/Mayo Clinic Lymphoma Specialized Program of Research Excellence, SPORE, Molecular Epidemiology Resource, MER, and Sylvester Comprehensive Cancer Center, University of Miami (UM) Miller School of Medicine, Miami, FL, USA) data

|  |  | **UM** | | | **MER** | | | **UM+MER** | |
| --- | --- | --- | --- | --- | --- | --- | --- | --- | --- |
| **Covariate** | **Status** | **Missing (n)** | **N** | **%** | **Missing (n)** | **N** | **%** | **N** | **%** |
| Total |  |  | 161 | 100 |  | 249 | 100 | 410 | 100 |
| Age (years) | >70 |  | 42 | 26.1 |  | 73 | 29.3 | 115 | 28 |
| Sex | M |  | 71 | 44.1 |  | 120 | 48.2 | 191 | 46.6 |
| ECOG | 2-4 |  | 11 | 6.8 | 5 | 20 | 8.2* | 31 | 7.7* |
| Stage | III-IV |  | 116 | 72.0 | 5 | 183 | 75.0* | 299 | 73.8* |
| Extranodal sites | >1 |  | 52 | 32.3 |  | 75 | 30.1 | 127 | 31 |
| Nodal sites | >4 |  | 27 | 16.8 | 147 | 15 | 14.7* | 42 | 16.0* |
| Symptoms | B | 11 | 39 | 26.0* |  | 39 | 15.7 | 78 | 19.5* |
| LDH | >UNL |  | 47 | 29.2 | 43 | 45 | 21.8* | 92 | 25.1* |
| ALC | <1 10^9^/L |  | 37 | 23.0 | 31 | 57 | 26.1* | 94 | 24.8* |
| Hemoglobin | <12 g/dL |  | 49 | 30.4 | 33 | 95 | 44.0* | 144 | 38.2* |
| Platelets | <100 10^9^/L |  | 18 | 11.2 | 17 | 23 | 9.9* | 41 | 10.4* |
| MZL subtype | SMZL/  ENMZL |  | 112 | 69.5 |  | 121 | 48.6 | 233 | 56.8 |
|  | NMZL/Diss. |  | 49 | 30.5 |  | 128 | 51.4 | 177 | 43.2 |
| ECOG: Eastern Cooperative Oncology Group; LDH: lactic dehydrogenase; ALC: absolute lymphocyte count; UNL: upper normal limit; SMZL: splenic marginal zone lymphoma; ENMZL: extranodal marginal zone lymphoma; NMZL: nodal marginal zone lymphoma; DissMZL: disseminated marginal zone lymphoma  *: percentage excluding missing.  **Note:** Missing values were considered as non-risk factor to define each index (MZL-IPI, IPI, FLIPI, and MALT-IPI). | | | | | | | | | |

**Table S5.** Progression-free survival by MZL-IPI of patients from the University of Iowa/Mayo Clinic Lymphoma Specialized Program of Research Excellence, SPORE, Molecular Epidemiology Resource, MER

|  | **PFS**  **events** | **N** | **%** | **5-yr PFS%**  **(95% CI)** | **HR**  **(95% CI)** | **p** |
| --- | --- | --- | --- | --- | --- | --- |
| All patients | 116 | 192 | 100 | 56 (49 – 63) | NA | NA |
| MZL-IPI score group: |  |  |  |  |  |  |
| Low (0) | 22 | 41 | 21.4 | 75 (59 - 86) | 1.00 (Ref.) | -- |
| Intermediate (1-2) | 68 | 113 | 58.9 | 52 (42 - 61) | 1.57 (0.97 - 2.54) | 0.069 |
| High (3-5) | 26 | 38 | 19.8 | 47 (30 - 62) | 2.04 (1.15 - 3.62) | 0.014 |
| High vs Inter. (ref) | -- | -- | -- | -- | 1.31 (0.83 - 2.05) | 0.249 |

HR: hazard ratio from Cox regression; p: p-value from Wald’s test for H0: HR=1.

Harrell’s C-index (SE) = 0.580 (0.024); Uno’s C-index (SE) = 0.563 (0.027)

**Table S6.** Progression-free survival by MZL-IPI of patients from the Sylvester Comprehensive Cancer Center, University of Miami Miller School of Medicine, Miami, FL, USA

|  | **PFS**  **events** | **N** | **%** | **5-yr PFS%**  **(95% CI)** | **HR**  **(95% CI)** | **p** |
| --- | --- | --- | --- | --- | --- | --- |
| All patients | 76 | 161 | 100 | 60 (52 – 68) | NA | NA |
| MZL-IPI score group: |  |  |  |  |  |  |
| Low (0) | 24 | 53 | 32.9 | 63 (47 - 75) | 1.00 (Ref.) | -- |
| Intermediate (1-2) | 33 | 79 | 49.1 | 66 (53 - 76) | 1.04 (0.61 - 1.76) | 0.885 |
| High (3-5) | 19 | 29 | 18.0 | 39 (20 - 59) | 2.16 (1.18 - 3.96) | 0.013 |
| High vs Inter. (ref) | -- | -- | -- | -- | 2.08 (1.18 - 3.66) | 0.011 |

HR: hazard ratio from Cox regression; p: p-value from Wald’s test for H0: HR=1

Harrell’s C-index (SE) = 0.567 (0.033); Uno’s C-index (SE) = 0.579 (0.060)

**Table S7.** Comparison of Hazard rations among MZL-IPI risk groups for all patients, patients treated with immunochemotherapy and patients treated with immunochemotherapy and Rituximab alone

|  | **All (n=456)** | **R-CHT (n=374)** | **RCHT+R mono (n=418)** |
| --- | --- | --- | --- |
| **Score** | **HR (95%CI** | **HR (95%CI)** | **HR (95%CI** |
| **Low** | 1.00 | 1.00 | 1.00 |
| **Internediate** | 2.30 (1.39-3.80) | 2.09 (1.21-3.64) | 2.29 (1.35-3.90) |
| **High** | 5.41 (3.13-9.38) | 4.60 (2.49-8.50) | 5.14 (2.84-9.28) |
| **High vs Intermediate** | 2.35 (1.60-3.45) | 2.20 (1.41-3.43) | 2.24 (1.47-3.41) |

**Table S8**. Cox PH model with covariates in continuous form

Model considering the covariates in continuous form: log(LDH/ULN LDH), log(ALC), Hemoglobin scaled by 12 g/dL and platelets with functional form (PLT/100)^-1^.

| **Covariate** | **HR (95%CI)** | **p-value** |
| --- | --- | --- |
| log(LDH/ULN LDH) | 1.68 (1.13-2.50) | 0.010 |
| log(ALC) | 0.73 (0.61-0.88) | 0.001 |
| Hb-12 | 0.84 (0.78-0.91) | <0.001 |
| (Platelets/100)^-1^ | 1.43 (1.10-1.85) | 0.008 |
| Histology NMZL/Diss | 1.64 (1.16-2.31) | 0.005 |

log: natural logarithm form (or base *e*); LDH: lactate dehydrogenase; ULN: upper limit of normality;

ALC: absolute lympcyte count 10^9^/L; PLT: platelets 10^9^/L.

Functional relation of platelets count related with log(HR):

**Supplementary Figures**

**Figure S1.** Flow-chart of patients with indolent non-follicular lymphoma enrolled in NF10 study of Fondazione Italiana Linfomi

Abbreviations: ENMZL: extranodal marginal zone lymphoma; SMZL: splenic marginal zone lymphoma; NMZL: nodal marginal zone lymphoma; DissMZL: disseminated marginal zone lymphoma; INFL: indolent nonfollicular lymphomas; RT: radiotherapy; W&W: watch and wait

**Figure S2. a)** Progression-free survival for all 501 MZL treated patients; **b)** Progression-free survival for patients treated immediately after diagnosis or after progression from watch and wait

**Figure S3.** Overall survival for all 501 MZL patients

**Figure S4. a)** Progression-free survival by subtype of marginal zone lymphoma in all 790 patients; **b)** Progression-free survival by subtype of marginal zone lymphoma in the 501 treated patients considered for model definition

**Figure S5 .** Progression-free survival stratified by the 6 (0 to 5) factors of the prognostic score

**Figure S6.** Progression-free survival of patients with marginal zone lymphoma according to MZL-IPI risk category (low, intermediate, high) of patients from the University of Iowa/Mayo Clinic Lymphoma Specialized Program of Research Excellence, SPORE, Molecular Epidemiology Resource, MER

**Figure S7.** Progression-free survival of patients with marginal zone lymphoma according to MZL-IPI risk category (low, intermediate, high) of patients from Sylvester Comprehensive Cancer Center, University of Miami Miller School of Medicine, Miami, FL, USA
